# Intra-Session Reliability of Sprint Performance on a Non-Motorised Treadmill for Healthy Active Males and Females

**DOI:** 10.5114/jhk/163180

**Published:** 2023-07-15

**Authors:** Kenji Doma, Jonathan D. Connor, Fabio Y. Nakamura, Anthony S. Leicht

**Affiliations:** 1 Sport and Exercise Science, James Cook University, Townsville, Queensland, Australia.; 2 Research Center in Sports Sciences, Health Sciences and Human Development (CIDESD), University of Maia (ISMAI), Maia, Portugal.

**Keywords:** running, power, speed, sex, measurement error, minimal detectable change

## Abstract

This study examined the intra-session reliability of sprint performance on a non-motorized treadmill amongst healthy, active male and female adults. One hundred and twenty participants (males n = 77; females n = 45) completed two familiarization sessions, followed by a third session that consisted of three trials (T1, T2, T3) of maximal sprints (4-s), interspersed by three minutes of recovery. Combining males and females exhibited moderate-to-excellent test-retest reliability (intra-class correlation coefficient, ICC), minimal measurement error (coefficient of variation, CV) and trivial differences between trials (effect size, ES) for speed, power, total work and acceleration (ICC = 0.82–0.98, CV = 1.31–8.45%, ES = 0.01–0.22). The measurement error was improved between comparisons of T1 vs. T2 (CV = 1.62–8.45%, ES = 0.12–0.22) to T2 vs. T3 (CV = 1.31–6.56%, ES = 0.01–0.07) and better for females (CV = 1.26–7.94%, ES = 0.001–0.26) than males (CV = 1.33–8.53%, ES = 0.06–0.31). The current study demonstrated moderate-to-excellent reliability and good-moderate measurement error during a 4-s sprint on a non-motorized treadmill. However, sex had a substantial impact with females exhibiting better values. Practitioners should employ at least two separate trials within a session, in addition to multiple familiarization sessions, to achieve reliable non-motorized treadmill sprint performances.

## Introduction

Sprint running is recognized as one of the most fundamental tasks for various team sports with athletes undertaking several sprint tasks during a match (Haugen and Buchheit, 2016). Accordingly, the kinematic (i.e., acceleration and speed) and kinetic (i.e., power) characteristics of short (e.g., ~4 s), maximal sprint performance are regularly assessed to monitor training adaptations and/or levels of fatigue (Cormier et al., 2020; Doma et al., 2018, 2021). Sprint performance is commonly assessed on the field using timing gate systems to ensure ecological validity; however, it lacks measures of anaerobic power and is significantly influenced by the local environment (Girard et al., 2015; Moinat et al., 2018). Recently, non-motorized treadmills (NMT) have been developed allowing athletes to complete tethered running in a laboratory-controlled environment and execute movement patterns more representative of sports (Highton et al., 2012; Hopker et al., 2009; Hughes et al., 2006). These treadmills are typically fitted with a force transducer connected to the athlete’s waist that records force, velocity and power performance (Tong et al., 2001). Measures derived from the NMT have been well correlated with common measures of field-based sprint performance, vertical jump and lower body muscular strength (Andre et al., 2013; Highton et al., 2012), demonstrating the utility of NMT for athletes’ monitoring.

Although the sprint task is favorable to replicate movement demands in sports, conducting sprint tests on a NMT may introduce variability across separate trials, given the distinct biomechanical movement patterns induced by the tethered system (Montgomery et al., 2016). Thus, familiarization trials are commonly incorporated to minimize learning effects with improved performance and reliability reported across a greater number of familiarization trials (Hopker et al., 2009; McLain et al., 2015; Tofari et al., 2015). However, most studies have examined the reliability of NMT sprint performance across separate days, and rarely within the same testing session. Determining the reliability of NMT sprint performance within a testing session will provide insight into the number of trials required to stabilize the performance metrics.

As far as we are aware, Tong et al. (2001) is the only study that examined intra-session reliability of sprint performance on a NMT with acceptable measurement error identified. However, this study along with other reliability studies (Highton et al., 2012; Hopker et al., 2009) have incorporated a modest sample size (i.e., <40 participants). A Delphi study examining the quality of reliability studies recommended that a sample size of at least 100 participants should be employed (Terwee et al., 2012), given that sample size influences the level of precision of reliability estimates (Polit, 2014). Furthermore, none of the studies to date have examined sex influences on the reliability of NMT-derived sprint performance, possibly due to the limited sample size employed. Given the increasing participation of, and call for, females in team-sports and reported sex differences in sprint performance (Leicht et al., 2011; Weber et al., 2006; Zupan et al., 2009), a greater understanding of the reliability of NMT-driven sprint performance for females is needed. Therefore, the aims of this study were to examine: 1) intra-session reliability of NMT-driven sprint performance measures; and 2) impact of sex on the reliability of NMT-driven sprint performance measures.

## Methods

### Participants

One hundred and twenty (age 22 ± 5 years; body height 175.4 ± 9.2 cm; body mass 73.8 ± 14.1 kg), physically active university males (n = 77; age 22 ± 5 years; body height 180.4 ± 6.8 cm; body mass 80.1 ± 12.7 kg) and females (n = 43; age 22 ± 5 years; body height 166.4 ± 5.4 cm; 62.5 ± 8.0 kg) were recruited for the study. None of the participants reported illness, injury or medication that would contraindicate any procedures. All participants had been undertaking moderate-to-high intensity exercise at least twice a week for 6 months or more with experience in short, sprint tasks through team sports. To maximize performance, participants refrained from high intensity exercise for at least 24 h prior to testing, and from consuming caffeine and food intake for at least 2 h prior to the testing session. Each participant provided written informed consent prior to the study commencement with all procedures approved by the James Cook University Research Ethics Committee (H6267; H7515) and conducted in accordance with the Declaration of Helsinki.

### Sprint Protocol

Prior to the sprint protocol, participants completed a warm-up equivalent to that of the familiarization sessions. The sprint protocol involved three maximal sprint trials for 4 s, interspersed by 3 min of passive rest intervals between each attempt. From each trial, the greatest and mean values for speed and power, along with acceleration within the first second, and total work for each trial were determined. Each sprint trial commenced from a standing, split-stance position whilst holding onto the NMT support bars. Participants were instructed to avoid pacing and accelerate to maximal sprint speed as fast as possible, with verbal encouragement provided. During each sprint, a non-elastic tether was connected between a belt around the participant’s waist and the NMT mounted force transducer sampling at a rate of 100 Hz. The height of the force transducer was adjusted so that the tether was parallel to the floor when participants adopted a sprinting position, which was established during the familiarization sessions.

### Design and Procedures

This study was carried out across three weeks with sprint testing conducted on the Woodway Force 3.0 NMT (Woodway, Waukesha, WI, USA) on three separate days. During the initial weeks, two practice sessions were conducted to familiarize participants with sprinting on the NMT, by completing three–four maximal sprints interspersed by self-selected recovery periods. Prior to the sprint protocol, participants completed warm-up exercises on a motorized treadmill (TM 601, Trackmaster, Newton, USA) that involved the following: jogging at 70% of the age-predicted heart rate for 3 min maximum; dynamic stretches of the lower extremity for 2 min; three bouts of 4-s sprints performed at slower speeds for females (13 km/h, 15 km/h and 15 km/h, respectively) than males (15 km/h, 17 km/h and 17 km/h, respectively), interspersed by 30-s rest intervals; and one bout of a 4-s maximal sprint after 3 min of passive rest. During the last testing session, participants completed three trials (T1, T2 and T3) of 4-s sprints, which were utilized to report on intra-day, test-retest reliability, for the entire group and each sex.

### Statistical Analyses

The measure of central tendency and dispersion for all data are reported as mean ± standard deviation (SD). All data were analyzed using IBM SPSS Statistics (version 27, IL, USA). A paired *t*-test was conducted to compare NMT-derived measures between trials (T1 vs. T2 and T2 vs. T3) with the level of significance (*p*) set at 0.05. To determine the magnitude of difference between trials, effect size (ES) was calculated using Cohen’s *d*, and the values were interpreted as follows: <0.20, 0.20–0.59, 0.60–1.19, 1.20–1.99, 2.00–4.00 and >4.00, rated as trivial, small, moderate, large, very large and extremely large effects, respectively (Hopkins, 2015). The level of agreement between trial results was determined via bias and limits of agreement (LOA), whilst measurement error was ascertained using the coefficient of variation (CV) with associated 95% confidence intervals (CI). For CV results, values of <5%, 5–9.9% and >10% were considered good, moderate and poor, respectively (Duthie et al., 2003). The test-retest reliability of the NMT-derived measures was assessed using intra-class correlation coefficients (single-rating, absolute-agreement, ICC_3,1_, 2-way mixed-effects model). The level of reliability was determined based on the 95% CI estimates of ICC with the following classifications employed: excellent (>0.90), good to excellent (0.75–1.00), good (0.75–0.90), moderate to good (0.50–0.90), moderate (0.50–0.75), poor to moderate (0–0.75) and poor (<0.50) (Koo and Li, 2016). For example, if the 95%CI of the ICC of a variable ranged from 0.92 to 0.99, then these values were considered excellent reliability. However, if the 95%CI of the ICC of a variable ranged from 0.88 to 0.92, then this was classified as good to excellent. The minimal detectable change (MDC) was also calculated by determining the typical error (standard deviation ÷ 2^0.5^), then using the following formula: typical error × 1.96 × 2^0.5^ (Teske et al., 2021).

## Results

For the total sample, all measures were significantly greater at T2 when compared to T1 (Table 1; trivial to small ES). Furthermore, mean speed, mean power and total work were significantly greater at T3 compared to T2 (trivial ES) with no differences in values of peak speed, peak power and acceleration between T2 and T3 (Table 1). Both males and females exhibited significantly greater peak speed, mean speed, mean power and total work at T2 compared to T1 (trivial to small ES) with peak power similar between trials (Table 1). Acceleration was significantly greater at T2 compared to T1 for males only (small ES; Table 1). For males, mean speed, mean power and total work were significantly greater at T3 than T2 (trivial ES), with no differences for peak speed, peak power and acceleration between these trials. No differences were evident for any of the variables between T2 and T3 for females (Table 1). The measurement error (CV) of all cohorts was good (1.26–4.45%) for all variables across all comparisons except for peak power and acceleration which was moderate (5.63–8.53%; Table 1). Generally, both measurement error and MDC values of all cohorts were smaller during the T2 vs. T3 evaluation when compared to the T1 vs. T2 evaluation. When comparing results between sexes, the measurement error and MDC values were mostly smaller (~5–50%) for females than males regardless of trial comparison. For example, the MDC values for PP was 340.92 W for females, which was almost half of the males with 605.94 W between T2 an T3.

**Table 1:** Mean ± standard deviation, effect size (ES) coefficient of variation (CV), and minimal detectable change (MDC) during each of the three trials for the total sample, male and female participants.

	T1	T2	T3	ES		CV (95 % CI)			MDC		
				T1 vs. T2	T2 vs. T3		T1 vs. T2	T2 vs. T3		T1 vs. T2	T2 vs. T3
**Total sample**											
PS (km/h)	19.0 ± 2.4	19.3 ± 2.5 ^b^	19.4 ± 2.5	−0.12^*^	−0.02^*^		1.62 (1.35, 1.88)	1.31 (1.11, 1.51)		1.02	0.87
PP (W)	2037.0 ± 594.3	2109.5 ± 648.5 ^a^	2079.1 ± 588.2	−0.12^*^	0.05^*^		8.17 (6.97, 9.37)	6.56 (5.49, 7.64)		612.2	526.7
MS (km/h)	15.0 ± 2.0	15.3 ± 2.0 ^b^	15.5 ± 2.0 ^c^	−0.18^*^	−0.07^*^		2.43 (2.02, 2.85)	1.62 (1.23, 2.00)		1.24	1.00
MP (W)	767.8 ± 207.3	797.2 ± 212.6 ^b^	809.8 ± 217.8 ^c^	−0.14^*^	−0.06^*^		3.92 (3.16, 4.68)	2.78 (2.16, 3.39)		118.1	91.2
TW (J)	3071.2 ± 829.1	3188.9 ± 850.2 ^b^	3239.4 ± 871.3 ^c^	−0.14^*^	−0.06^*^		3.92 (3.16, 4.68)	2.78 (2.16, 3.39)		472.5	365.0
Acc (m∙s^-2^)	2.71 ± 0.62	2.85 ± 0.6 ^b^	2.86 ± 0.6	−0.22^†^	−0.01^*^		8.45 (6.92, 9.99)	5.92 (4.65, 7.20)		0.74	0.62
**Male participants**											
PS (km/h)	20.5 ± 1.4	20.8 ± 1.4 ^b^	20.9 ± 1.2	−0.26^†^	−0.06^*^		1.67 (1.32, 2.01)	1.33 (1.09, 1.56)		1.10	0.95
PP (W)	2349.3 ± 473.1	2429.6 ± 545.2	2382.7 ± 462.7	−0.16^*^	0.09^*^		7.80 (6.44, 9.15)	6.93 (5.68, 8.18)		698.51	605.94
MS (km/h)	16.1 ± 1.3	16.5 ± 1.3 ^b^	16.7 ± 1.1 ^d^	−0.31^†^	−0.17^*^		2.68 (2.11, 3.25)	1.83 (1.33, 2.32)		1.44	1.13
MP (W)	886.8 ± 150.8	922.7 ± 148.1 ^b^	942.5 ± 140.5 ^d^	−0.24^†^	−0.14^*^		4.45 (3.45, 5.46)	3.26 (2.50, 4.03)		140.06	106.21
TW (J)	3547.3 ± 603.1	3690.7 ± 592.2 ^b^	3769.9 ± 561.9 ^d^	−0.24^†^	−0.14^*^		4.45 (3.45, 5.46)	3.26 (2.50, 4.03)		560.24	424.85
Acc (m∙s^-2^)	3.00 ± 0.55	3.16 ± 0.54 ^b^	3.20 ± 0.44	−0.29^†^	−0.10^*^		8.53 (6.81, 10.26)	6.27 (4.62, 7.92)		0.83	0.70
**Female participants**											
PS (km/h)	16.4 ± 1.4	16.6 ± 1.3 ^a^	16.6 ± 1.3	−0.14^*^	−0.001^*^		1.59 (1.21, 1.97)	1.26 (0.99, 1.54)		0.85	0.72
PP (W)	1477.8 ± 310.8	1536.3 ± 359.9	1535.5 ± 348.5	−0.18^*^	0.002^*^		7.94 (6.14, 9.75)	5.90 (4.61, 7.19)		421.51	340.92
MS (km/h)	12.9 ± 1.2	13.2 ± 1.2 ^b^	13.2 ± 1.2	−0.23^†^	−0.01^*^		2.08 (1.58, 2.59)	1.39 (1.07, 1.71)		0.77	0.65
MP (W)	554.7 ± 90.1	572.6 ± 88.5 ^b^	572.3 ± 93.4	−0.20^†^	0.003^*^		3.43 (2.61, 4.25)	2.42 (1.82, 3.03)		56.77	46.51
TW (J)	2218.7 ± 360.5	2290.3 ± 353.8 ^b^	2289.3 ± 373.4	−0.20^†^	0.003^*^		3.43 (2.61, 4.25)	2.42 (1.82, 3.03)		227.07	186.02
Acc (m∙s^-2^)	2.20 ± 0.34	2.30 ± 0.40	2.24 ± 0.37	−0.26^†^	0.16^*^		7.33 (5.35, 9.31)	5.63 (4.28, 6.99)		0.55	0.45

T1 – Trial 1; T2 – Trial 2; T3 – Trial 3; CI – confidence interval; PS – peak speed; PP – peak power; MS – mean speed; MP – mean power; TW – total work; Acc – acceleration during 1^st^ second; km – kilometer; h – hour; W – watts; J – joules; m – meter; s – second

a *p* < 0.05, ^b^ *p* < 0.01 vs. T1; ^c^ *p* < 0.05, ^d^ *p* < 0.01 vs. T2; ^*^ trivial, ^†^ small

**Figure 1:**
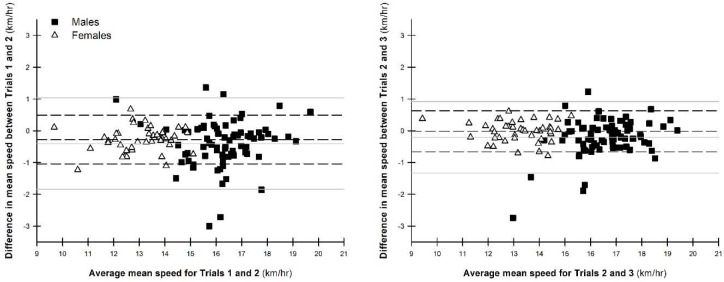
Bland-Altman plots demonstrating the level of agreement for mean speed between Trial 1 vs. Trial 2 and Trial 2 vs. Trial 3 for males and females. Grey solid lines – males; black dashed lines – females

The test-retest reliability of the total sample was excellent for all measures across all comparisons (ICC = 0.93–0.99), except peak power and acceleration was good to excellent (ICC = 0.75–0.93; Table 2). For males, the test-retest reliability for peak speed was excellent between T2 vs. T3 (ICC = 0.90–0.96), whilst it was good to excellent for peak speed between T1 vs. T2 and mean speed, mean power and total work across all comparisons (ICC = 0.77–0.96; Table 2). The test-retest reliability for male peak power between T1 vs. T2, and acceleration across all comparisons was moderate to good (ICC = 0.57–0.88; Table 2). For females, peak speed, mean speed, mean power and total work exhibited excellent test-retest reliability across all comparisons (ICC = 0.90–0.98; Table 2). Furthermore, the test-retest reliability for peak power between T2 vs. T3 was good to excellent (Table 2). In contrast, the test-retest reliability for peak power during the T1 vs. T2 evaluation, and acceleration during the T1 vs. T2 and T2 vs. T3 evaluations was moderate to good (ICC = 0.53–0.90; Table 2). The level of agreement was generally better for all variables between T2 vs. T3 compared to T1 vs. T2 (Figure 1; Table 2). Furthermore, the level of agreement of all variables was better for females than males (Figure 1; Table 2).

**Table 2:** The intra-class correlations (ICC) and measurement bias with limits of agreement (LOA) for the total sample, male and female participants.

	ICC (95 % CI)		Measurement bias (+/- 95 % LOA)	
**Total sample**	T1 vs. T2	T2 vs. T3	T1 vs. T2	T2 vs. T3
PS (km/h)	0.98 (0.96–0.98)	0.98 (0.97–0.99)	−0.30 ± 1.02	−0.05 ± 0.87
PP (W)	0.87 (0.82–0.91)	0.91 (0.87–0.93)	−72.47 ± 612.16	30.35 ± 526.69
MS (km/h)	0.95 (0.93–0.97)	0.97 (0.96–0.98)	−0.36 ± 1.24	−0.14 ± 1.00
MP (W)	0.96 (0.94–0.97)	0.98 (0.97–0.98)	−29.41 ± 118.12	−12.62 ± 91.24
TW (J)	0.96 (0.94–0.97)	0.98 (0.97–0.98)	−117.65 ± 472.49	−50.47 ± 364.98
Acc (m∙s^-2^)	0.82 (0.75–0.87)	0.87 (0.82–0.91)	−0.14 ± 0.74	−0.01 ± 0.62
**Male participants**				
PS (km/h)	0.92 (0.87–0.95)	0.93 (0.90–0.96)	−0.35 ± 1.10	−0.08 ± 0.95
PP (W)	0.75 (0.64–0.84)	0.81 (0.72–0.88)	−80.24 ± 698.51	46.87 ± 605.94
MS (km/h)	0.85 (0.77–0.90)	0.89 (0.83–0.93)	−0.40 ± 1.44	−0.21 ± 1.13
MP (W)	0.89 (0.83–0.93)	0.93 (0.89–0.96)	−35.85 ± 140.06	−19.8 ± 106.21
TW (J)	0.89 (0.83–0.93)	0.93 (0.89–0.96)	−143.38 ± 560.24	−79.19 ± 424.85
Acc (m∙s^-2^)	0.70 (0.57–0.80)	0.74 (0.62–0.83)	−0.16 ± 0.83	−0.05 ± 0.70
**Female participants**				
PS (km/h)	0.95 (0.91–0.97)	0.96 (0.93–0.98)	−0.20 ± 0.85	−0.002 ± 0.72
PP (W)	0.80 (0.65–0.88)	0.88 (0.79–0.93)	−58.55 ± 421.51	0.77 ± 340.92
MS (km/h)	0.94 (0.90–0.97)	0.96 (0.93–0.98)	−0.28 ± 0.77	−0.02 ± 0.65
MP (W)	0.95 (0.91–0.97)	0.97 (0.94–0.98)	−17.89 ± 56.77	0.24 ± 46.51
TW (J)	0.95 (0.91–0.97)	0.97 (0.94–0.98)	−71.57 ± 227.070	0.95 ± 186.02
Acc (m∙s^-2^)	0.72 (0.53–0.84)	0.83 (0.70–0.90)	−0.10 ± 0.55	0.06 ± 0.45

T1 – Trial 1; T2 – Trial 2; T3 – Trial 3; CI – confidence interval; PS – peak speed; PP – peak power; MS – mean speed; MP – mean power; TW – total work; Acc – acceleration during 1^st^ second; km – kilometer; h – hour; W – watts; J – joules; m – meter; s – second

## Discussion

The current study identified good to excellent test-retest intra-session reliability with minimal measurement error for most of the NMT-derived variables for all cohorts. Furthermore, better measurement error, measurement bias, magnitude of differences and MDC were evident for all NMT-derived variables between T2 vs. T3 compared with the T1 vs. T2 evaluation. Compared to males, females demonstrated better reliability for most NMT-derived variables. These results encourage practitioners to employ at least two separate familiarization sessions, in conjunction with two maximal sprint trials during each testing session to achieve the greatest NMT results. Furthermore, practitioners should be aware of differences in reliability between sex when assessing NMT sprinting ability.

The current study demonstrated better intra-session reliability and measurement error of the NMT-derived variables between T2 vs. T3 than between T1 vs. T2, despite the incorporation of two familiarization sessions beforehand. It was difficult to compare our findings to previous studies, as we are the first to report on changes in intra-session reliability of sprint performance on a NMT across more than two trials. Nonetheless, our findings expand upon prior work that reported lower CV values for power output during a NMT sprint protocol between the 3^rd^ and 4^th^ testing days (3.2–10.1%) when compared to the 1^st^ and 2^nd^ (4.3–20.7%), and 2^nd^ and 3^rd^ (4.8–21.1%) testing days (Hopker et al., 2009; Tofari et al., 2015). Based upon our findings and those of others (Hopker et al., 2009; Tofari et al., 2015), we would recommend incorporating at least two familiarization sessions, in conjunction with two maximal trials during each testing session to optimize reliable short, sprint performance protocols on a NMT for future interventions.

In line with our recommendation, we focused on the T2 vs. T3 evaluation and observed moderate to excellent test-retest reliability and good-moderate measurement error for mean and peak speed, mean and peak power, total work and acceleration for all (total, male and female) cohorts. These results align with previous studies that examined the reliability of short (~6 s), NMT sprint protocols which reported moderate to excellent test-retest reliability for mean (ICC = 0.83–0.93) and peak power (ICC = 0.54–0.83) (Hopker et al., 2009), and moderate to good measurement error for peak speed (CV = 1.3%) (Tong et al., 2001) and mean power (CV = 7.4–8.2%) (Hopker et al., 2009; Tong et al., 2001). Accordingly, our findings support previous evidence indicating moderate to excellent test-retest reliability and acceptable measurement error for sprint speed and power generated from a NMT, albeit with a much larger sample size.

An interesting finding of the current study was that most NMT-derived measures demonstrated high levels of test-retest reliability, except for acceleration. To our knowledge, prior studies have not reported on the reliability of acceleration derived from a NMT protocol. Acceleration is calculated as the change in sprint speed from a stationary position and appears to be a more complex component of a sprint task (Haugenet al., 2019), which may contribute to greater variability. Regardless, the current study has broadened the understanding of NMT sprint protocol reliability with acceleration exhibiting the greatest improvement in reliability measures between comparisons (ICC = 0.82 to 0.87; CV = 8.45 to 5.92%; bias = −0.14 to −0.01). This improvement may be a consequence of participants becoming familiar with the frictional resistance of the tethered system at the commencement of the sprint task.

In the current study, small MDC values were evident for all cohorts that represented ~5% of the mean. For most of the other NMT variables, relatively low MDC values (~10% of mean) were exhibited for all cohorts except for peak power and acceleration where the MDC was comparatively larger (i.e. ~14–25% and 13–22% of the mean, respectively). The greater MDC values for peak power may be due to its instantaneous nature, whilst the complexity of the movement during the initiation of the sprint task may have caused greater MDC values for acceleration. Thus, the majority of the NMT-derived measures appeared to provide appropriate sensitivity to detect changes in response to exercise interventions (~5–10% changes in mean), although caution should be taken when interpreting differences in peak power and acceleration.

A novel finding of the current study was that the reliability (ICC, LOA, CV) and sensitivity (MDC) of all NMT-derived measures were greater for females than males. To our knowledge, no prior studies have compared the reliability of run-sprint performance measures between sexes, whether in a laboratory or field setting. Delextrat et al. (2015) speculated that females may have greater stability than men due to a lower stature, thereby translating run-sprint mechanics better during agility-related activities. Considering that the current females were of a lower stature than males, it is possible that this smaller stature may have resulted in consistent sprint mechanics of the NMT-derived measures. However, it is important to note that balance was not measured as part of the current study. Future biomechanical examination of NMT sprinting may confirm the origin of these sex differences in reliability. Nonetheless, the distinct MDC values reported in the current study may provide practitioners with useful information to support future sprint performance interventions for either sex, and when combined.

In conclusion, our study demonstrated that a short (4-s), sprint protocol on a NMT exhibited good to excellent intra-session reliability for most variables that was enhanced across three trials, namely between the 2^nd^ and the 3^rd^ one. Compared to males, females exhibited better reliability values possibly due to smaller stature. Practitioners are encouraged to employ at least two trials in the same session (in addition to multiple familiarization sessions) to achieve the greatest results via a reliable and reproducible short (4-s), NMT sprint protocol.
